# Efficacy of GnRH Pulses in Hypogonadism Secondary to Primary Empty Sella: Case Report

**DOI:** 10.1007/s43032-024-01637-1

**Published:** 2024-07-03

**Authors:** Chenxi Ran, Guiliang Peng, Rufei Shen, Qian Liao, Mingyu Liao, Qixian Wang, Ling Zhou, Hongting Zheng, Min Long

**Affiliations:** 1 Department of Endocrinology, The Second Affiliated Hospital, Xinqiao Hospital, Army Medical University, No.83 Xinqiao Zhengjie, Shapingba District, Chongqing, 400037 PR China; 2 Department of Endocrinology, The First Affiliated Hospital, Southwest Hospital, Army Medical University, No.30 Gaotanyan Zhengjie, Shapingba District, Chongqing, 400038 PR China

**Keywords:** Empty sella, Hypogonadotropic hypogonadism, Gonadotropin-releasing hormone pump, Pulse treatment, Sperm

## Abstract

This study aims to assess the effectiveness of pulsed gonadotropin-releasing hormone (GnRH) micropump replacement therapy in the treatment of hypogonadotropic hypogonadism (HH) caused by primary empty sella (PES).The efficacy of pulsed GnRH replacement therapy using the micropump was evaluated in a middle-aged male patient with HH who had experienced the loss of his only child. Relevant literature was also consulted to compare the differences between pulse GnRH treatment and conventional treatment in terms of the development of secondary sexual characteristics, sex hormone levels, sperm production rate, and sperm activity rate in male patient with HH.In this report, a 45-year-old male diagnosed with HH and PES presented with fatigue and decreased libido. The main characteristics included decreased follicle stimulating hormone (FSH) levels of 0.03 mIU/mL, luteinizing hormone (LH) levels of 0.02 mIU/mL, and testosterone (T) levels of 0.72 nmol/L. Magnetic resonance imaging (MRI) revealed an empty sella. Semen analysis showed a small number of normal sperm with reduced motility. During treatment with the micropump pulse GnRH, the patient experienced no side effects and showed improvements in fatigue, reduced libido, sexual urge, anxiety, and feelings of inferiority. LH, FSH, and T levels returned to normal, while sperm activity rate increased to 79.9%. Ultimately, the patient's spouse achieved a natural pregnancy.Pulsed gonadotropin delivery using the micropump demonstrates good efficacy and tolerability, and aligns more closely with the physiological rhythm of GnRH secretion in the human body.

## Introduction

Empty Sella (ES) is a radiological diagnosis characterized by the expansile dilation of the sella turcica, resulting in pituitary compression and partial or complete filling with cerebrospinal fluid (CSF). ES has a prevalence rate of 8% to 35% in the general population, predominantly observed in women aged 30 to 40 years [1]. ES is classified as primary empty sella (PES) and secondary empty sella (SES). The prevalence of hypopituitarism due to ES is estimated to range between 19 and 68% [2]. The growth hormone axis is most commonly affected, closely followed by the gonadotropic axis. Additionally, sporadic cases of hyperprolactinemia have also been documented [3, 4].

Hypogonadotropic hypogonadism (HH) is a disorder characterized by impaired testicular function caused by lesions in the hypothalamus or pituitary gland [5]. The presence of HH not only causes significant mental and psychological distress to patient and their families but also imposes a substantial burden on society. Androgen replacement therapy is an effective approach to maintaining male sexual function and secondary sexual characteristics [6]. Moreover, for individuals with reproductive aspirations, gonadotropin replacement has the potential to promote testicular growth, stimulate spermatogenesis, improve sperm motility, and facilitate fertility in selected male patient, offering hope for the realization of parenthood [5].

This study presents a case report of a male patient diagnosed with secondary HH and ES who underwent treatment with micropump pulse gonadotropin-releasing hormone (GnRH), resulting in a favorable therapeutic outcome. Additionally, a comprehensive review of the relevant literature was undertaken, comparing the findings with conventional approaches to HH management, providing valuable insights into the diagnosis and treatment of this condition.

### Case Presentation

A 45-year-old male was referred to our department in June 2021 due to a 3-month history of fatigue and decreased libido. The patient has a history of successful tuberculosis treatment and no other medical conditions. He has a smoking history of over 20 years, with a daily consumption of 15–20 cigarettes, and has never tried to quit. The patient has been married for over 20 years, but tragically lost his only son. He does not have any other children, and there is no significant family medical history.

Three months prior to admission, the patient experienced unexplained fatigue and hyposexuality, but unfortunately, his symptoms were not appropriately addressed, and no further treatment was provided. One month prior to admission, the patient developed tender bilateral breast enlargement without lactation. An MRI conducted at a local hospital revealed an ES, bilateral ethmoid sinusitis, and a cyst in the left maxillary sinus. Additionally, the ultrasound of the male reproductive system detected no abnormalities in the bilateral testicles, while a breast ultrasound confirmed gynecomastia. The results of visual acuity and visual field tests were within normal range. Sex hormone analysis showed elevated levels of prolactin (PRL) at 452.6 pmol/L (55.97–278.35 pmol/L), estradiol (E2) at 55.10 pg/mL (< 20–47 pg/mL), but T levels dropped to 0.58 noml/L (6.07–27.10 noml/L). The local hospital diagnosed the patient with "Empty Sella, Hyperprolactinemia" and prescribed Bromocriptine Mesylate tablets 2.5 mg once daily and Rubi tablets (a traditional Chinese medicine for promoting blood circulation and dispersing knot) 5 tablets thrice daily for a month, but no significant improvement in the mentioned symptoms were observed. Subsequently, the patient sought additional treatment at our hospital and was admitted with a diagnosis of "Empty Sella, Hypogonadism".

On admission, the patient underwent a physical examination that yielded the following measurements: height of 176 cm, weight of 69 kg, body temperature of 36.3 °C, pulse rate of 84 bpm, respiratory rate of 19 breaths per minute, and blood pressure of 113/81 mmHg. The patient had a beard and normal distribution of body hair, including abundant and dark underarm hair. Adam's apple was visible, and there was no sign of thyroid enlargement.

### Imaging Examination

**Fig. 1:**
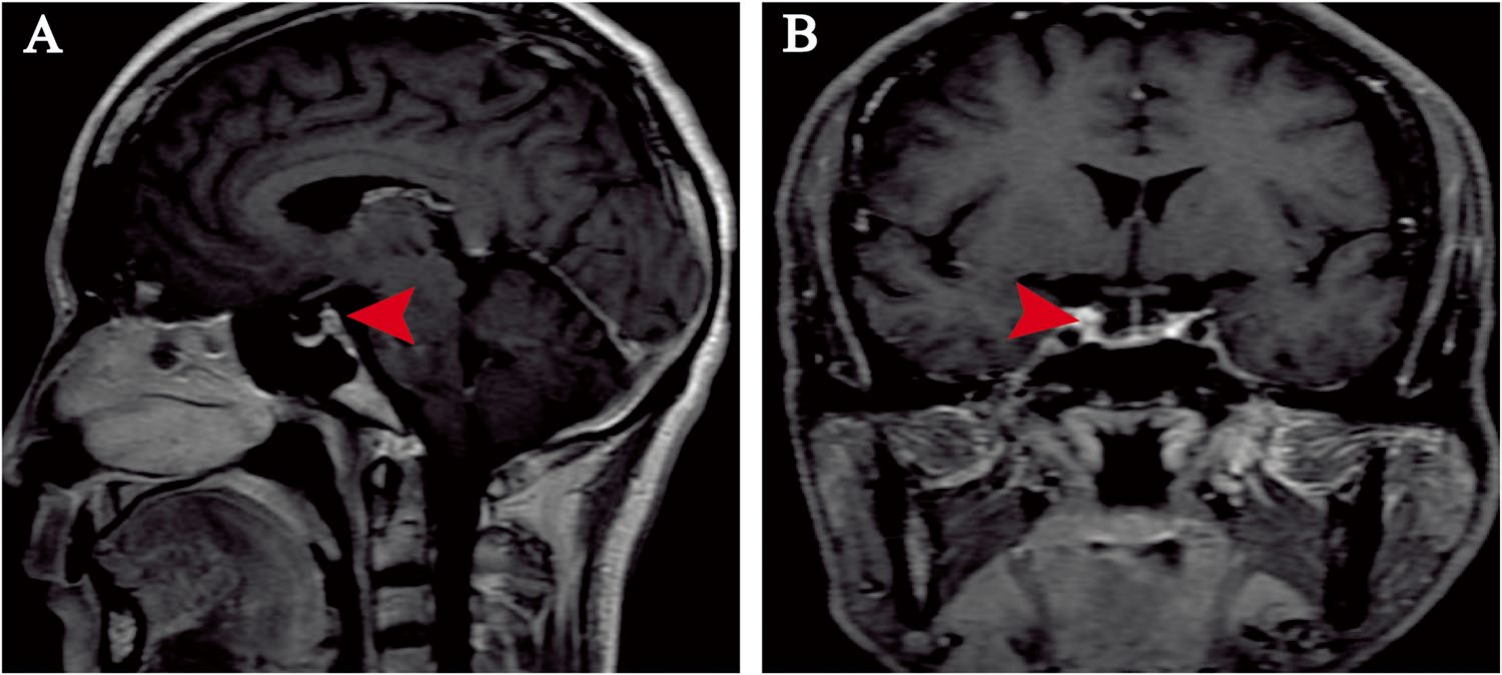
Magnetic resonance imaging (MRI) revealed a partially empty sella (arrow). **A**. Sagittal T1W. **B**. Coronal T1W

Reexamination of the sellar area by MRI in our hospital after admission revealed ES, the sella was not enlarged and the bottom of the sella was not sunken. The pituitary gland in the sellar region was thin, with a height of about 1.45 mm. The pituitary gland showed obvious uniform enhancement on enhanced scan. The rest of the sellar region was filled with CSF signal shadow (Fig. 1). Bilateral color Doppler ultrasound revealed bilateral development of the mammary glands. Additionally, the color Doppler scan of the male reproductive system revealed that the right testicle measured 3.9 cm in length and 1.8 cm in thickness. The right epididymal head had a thickness of 0.66 cm. Similarly, the left testicle measured 4.0 cm in length and 1.83 cm in thickness, with the left epididymal head having a thickness of 0.67 cm. Ultrasonography revealed no obvious abnormalities in the bilateral adrenal glands.

### Laboratory Examination

**Fig. 2:**
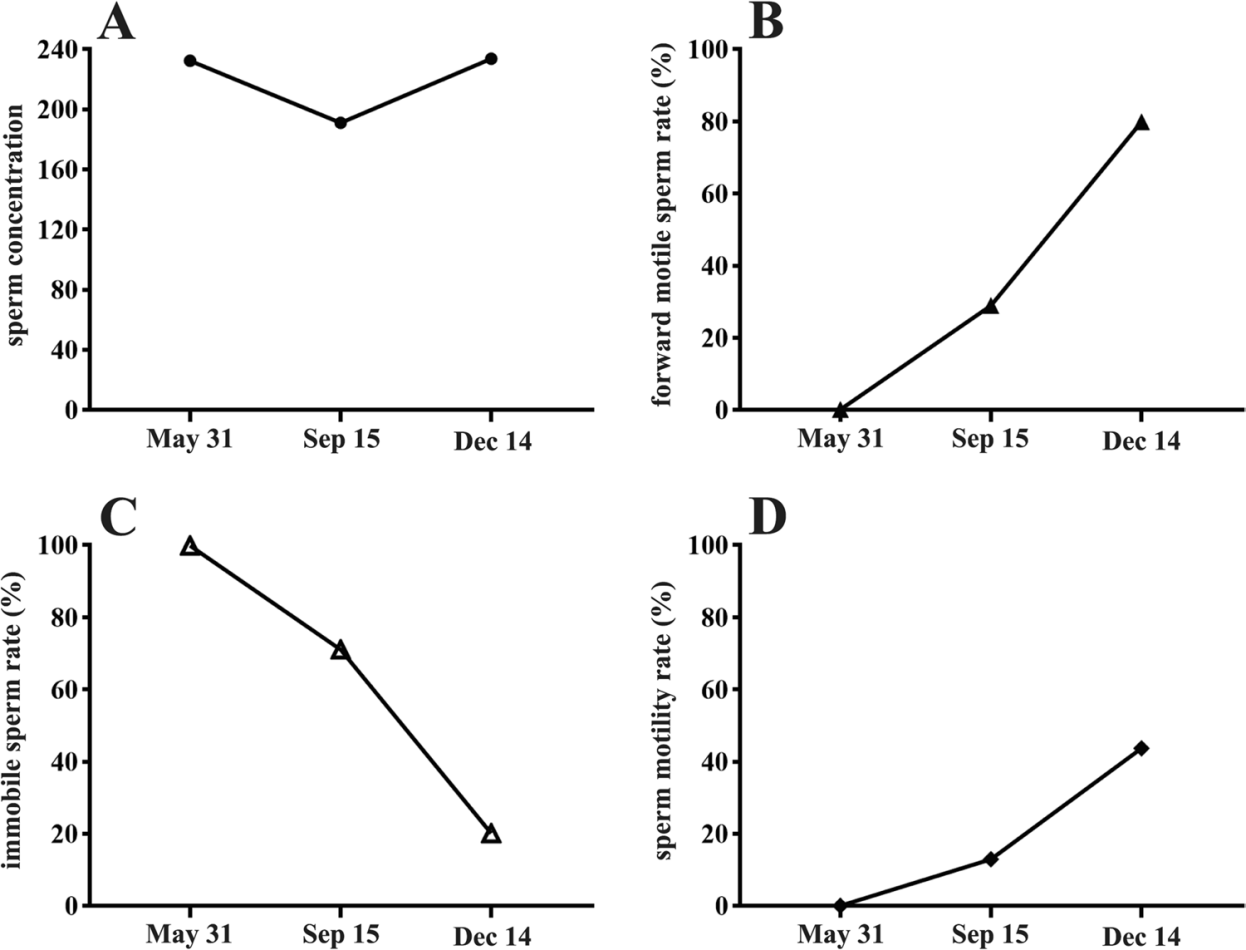
Sperm motility rate and semen concentration results. **A**. sperm concentration, **B**. the forward motile sperm rate (%), **C**. the immobile sperm rate (%), **D**. the sperm activity rate (%)

**Fig. 3:**
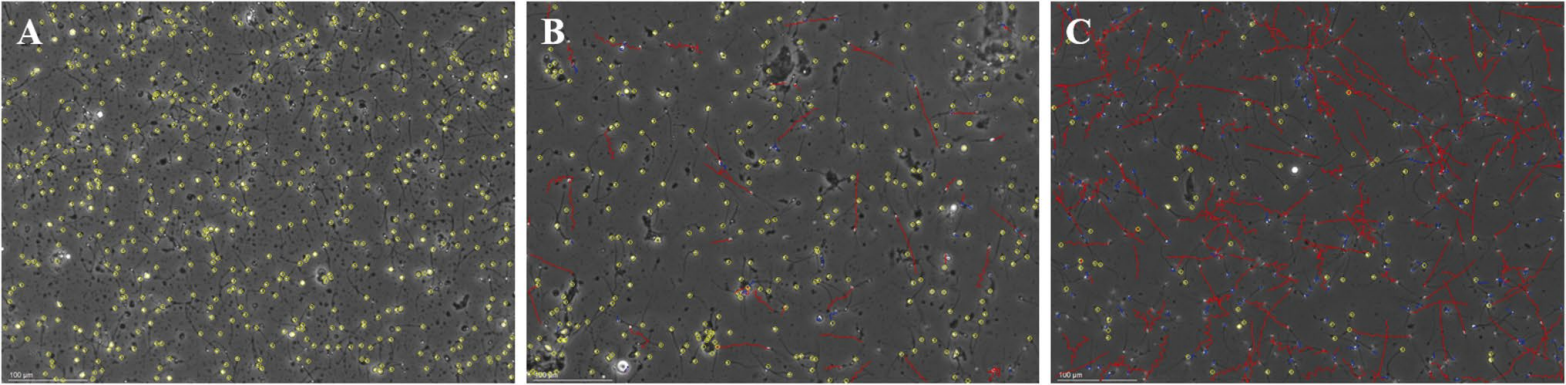
The status of progressive spermatozoa (purple), nonprogressive spermatozoa (red) and immobile sperm (yellow). **A**. pre-treatment, **B**. 3 months post-treatment, **C**. 6 months post-treatment

Endocrine function tests revealed decreased FSH levels of 0.03 mIU/mL (1.27–19.26 mIU/mL), LH 0.02 mIU/mL (1.24–8.62 mIU/mL), and T 0.72 nmol/L (Table 1). Other endocrine hormone levels were within the normal range: PRL 4.69 ng/mL (2.64–13.13 ng/mL), E2 11.00 pg/mL (<20–47 pg/mL), progesterone (P) 0.81 pg/ml (0.14–2.06), sex hormone-binding globulin (SBHG) 133.0 nmol/L (34.3–147 nmol/L), dehydroepiandrosterone (DHEA) 208.7ug/dL (139.7–484.4 ug/dL); thyroid-stimulating hormone (TSH) 1.23 mU/L (0.27–4.2 mIU/L), free triiodothyronine (FT3) 4.59 pmol/L (3.1–6.89 pmol/L), and free thyroxine (FT4) 13.49 pmol/L (11–22 pmol/L); adrenocorticotropic hormone (ACTH) 68.47 ng/mL (7.2–63.3 ng/L), cortisol 606.3 nmol/L (66–579.4 nmol/L); growth hormone (GH) 1.35ug/L (0–8 ug/L) and insulin-like growth factor-1 (IGF-1) 116 ng/mL (115–307 ng/mL). Blood count, blood glucose, glycosylated hemoglobin, liver function, and renal function were all normal. The GnRH stimulation test (gonarelin 100 mg i.v.) was successfully performed: The peak LH value was more than three times the baseline value (0.11 IU/L to 0.46 IU/L), with a peak at 60 min. Similarly, peak FSH was more than double baseline (0.05 to 0.12 IU/L), peaking at 120 min. Human chorionic gonadotropin (HCG) stimulation test (HCG 2000U i.m.) was performed: The peak level of T increased to more than twice the value at baseline (0.5 ng/mL to 8.4 ng/mL) and peaked at 72 h. Semen analysis was performed: The sperm concentration (million/mL) was measured as 232.3 (normal: ≥ 15), the forward motile sperm rate (%) was recorded as 0 (normal: ≥ 32%), the immobile sperm rate (%) was 99.9, and the sperm activity rate (%) was found to be 0.1 (normal: ≥ 40%, Fig. 2). And the majority of sperm are in a dormant state (Fig. 3A).

**Table 1:** Pre and post-treatment sex hormone survey

	LH (mIU/ml)	FSH (mIU/ml)	E2 (pg/ml)	P (pg/ml)	T (nmol/L)	PRL (ng/ml)
Pre-treatment	0.02	0.03	11	0.81	0.72	4.69
1 month after treatment	2.54	1.52	< 10	0.2	4.37	8.54
3 month after treatment	6.85	3.76	< 10	0.1	22.08	8.44
6 month after treatment	9.37	2.82	31	0.2	23.39	9.23

Abbreviation, hormone (reference range): E2 estradiol (< 20–47 pg/mL); FSH follicular stimulating hormone (1.27–19.26 mIU/mL); LH luteinizing hormone (1.24–8.62 mIU/mL); P progesterone (0.14–2.06 pg/ml); PRL prolactin (2.64–13.13 ng/ml); T testosterone (6.07–27.10 noml/L)

### Diagnosis and Treatment

Diagnostic considerations were made based on these findings. The patient was ultimately diagnosed with ES and hypogonadotropic hypogonadism. The patient's desire for a possible second birth prompted a consultation with a neurosurgeon to assess the feasibility of the surgical intervention, despite its limited potential benefit. Consequently, the patient was prescribed oral tamoxifen at a daily dosage of 20 mg to reduce breast development, and received pulsed gonadotropin (Maanshan FengYuan Pharmaceutical Co., LTD., Anhui, China) infusion using a micropump (Shanghai Minimally Invasive Life Technology Co., LTD., Shanghai, China). The micropump was administered a 10 μg dose within 1 min every 90 min.

After 1 month of treatment initiation, LH, FSH, and T levels returned to normal (Table 1), accompanied by a gradual improvement in fatigue and hyposexuality symptoms. Following 3 months of treatment, tamoxifen was discontinued, and the semen analysis displayed a sperm concentration of 191.1 million/ml, along with a forward motile sperm rate of 12.9%, an immobile sperm rate of 71%, and a motile sperm rate of 29% (Fig. 2). And Partial immobile sperm transition to progressive sperm (Fig. 3B). The treatment with a GnRH pulse (10 ug/90 min) was continued. During the six-month follow-up, semen analysis demonstrated additional improvements, including a sperm concentration of 233.8 million/ml; a forward motility rate of 43.7%, an immobile sperm rate of 20.1%, and a motility rate of 79.9% (Fig. 2). There is a significant decrease in the proportion of immobile sperm and a significant increase in the proportion of progressive sperm (Fig. 3C). Notably, normal sperm count did not change significantly. The GnRH pulse treatment (10 μg/90 min) was continued. The patient's wife successfully conceived naturally after 9 months of treatment. Subsequently, after 10 months of treatment, the GnRH pump was discontinued, and maintenance androgen replacement therapy was initiated.

## Discussion

The diagnosis of ES syndrome is typically made using radiological techniques such as computed tomography (CT) or MRI. Partial ES is characterized by the presence of CSF filling less than 50% of the sella turcica. Complete ES is defined as more than 50% of the sella being occupied by CSF, with pituitary tissue measuring ≤ 2 mm [7]. PES is often discovered incidentally in asymptomatic patients, but it can also present with various degrees of neurological, visual, and/or endocrine dysfunction. De Marinis et al. observed 213 cases of PES and found evidence of endocrine dysfunction in 19% of patients, with only 8% exhibiting hormonal deficiency [8]. However, a recent multicenter study involving 402 patients with PES reported a prevalence of hypopituitarism of 40.5%, with hypogonadism (20.4%) being the most common [2].

Extensive research is focused on the nexus between arcuate kisspeptin neurons and GnRH pulsatility. Kirilov et al.[9] highlighted the hypothalamic kisspeptin signaling pathway's role in the production of pulsatile human gonadotropins. Clarkson et al. revealed a population of hypothalamic kisspeptin neurons represents the gonadotropin-releasing hormone (GnRH) pulse generator. A recent Japanese study [10] provides direct evidence that KNDy neurons act as GnRH pulse generators, demonstrating that at least 20% of these neurons are adequate for sustaining follicular and spermatogenic function via GnRH pulsation, underpinning fertility in both sexes. Concurrently, other research [11] has revealed that neurokinin B, a molecule expressed in kisspeptin neurons, enhances GnRH pulsatile neural activity in goats when locally injected into the arcuate nucleus. Furthermore, human males with mutations in the kisspeptin signaling pathway have achieved successful reproduction following exogenous hormone therapy [12]. A high level of PRL inhibits kisspeptin neuronal expression, thereby reducing gonadotropin levels [13], which may also account for the compromised GnRH pulsatility in this individual.

In this particular case, the patient had no previous history of hypogonadism and exhibited normal growth and development. A comprehensive physical examination conducted by a specialist revealed normal development of the external genitalia. Laboratory tests indicated low levels of testosterone, FSH, and LH. An MRI of the sellar region showed evidence of an ES. Following the unexpected loss of his son, this middle-aged male patient expressed a strong desire to have another child. However, in patient with HH, reduced production of gonadal hormones (LH, FSH) results in the absence of secondary sexual characteristics, decreased testicular size, penile shortening, diminished erectile capacity, sexual dysfunction, and other reproductive dysfunctions [14]. These effects can contribute to the development of low self-esteem and depression in patients, not only impacting their daily lives and relationships but also having negative implications for their families and society as a whole. Therefore, it is crucial to correct hypogonadism and stimulate sperm production. Prior to initiating treatment, it is necessary to assess the secretory status of the pituitary–gonadal axis and the functional integrity of the testicular interstitial cell reserve.

The dynamic stimulation test is commonly employed to assess the secretion status of gonadotrophins and the function of testicular interstitial cells. The GnRH stimulation test is widely used for evaluating the reserve function of pituitary gonadotropin cells and the excitatory state of the hypothalamic-pituitary–gonadal (HPG) axis. In more than 95% of normal adult males, the GnRH stimulation test results in an increase in LH levels of 2–3 times above basal, with a peak higher than 12 U/L and a peak time between 15–30 min [15]. Wu XY et al. proposed that patients with a peak LH level below 4 U/L are more likely to be diagnosed with HH and will require lifelong androgen replacement therapy [16]. In this case, basal levels of T, LH, and FSH were all low. Following the GnRH stimulation test, LH levels increased more than 3 times compared to the basal level, indicating a well-functioning pituitary gonadotropin reserve. However, the peak LH level remained significantly low, supporting the diagnosis of "hypogonadotropic hypogonadism." The peak value of T was more than double the baseline value, suggesting good reserve function of the testicular Leydig cells based on the HCG stimulation test. The relationship between the results of the GnRH or HCG stimulation tests and the subsequent treatment regimen is currently underreported and requires further discussion.

The objective of pharmacological treatment is to address the physiological and reproductive needs of patients with HH. Exogenous androgen supplementation alone is limited to maintaining male sexual function and secondary sexual characteristics without promoting sperm production. GnRH therapy aligns better with the physiological regulatory model of the HPG axis in humans, facilitating genital development and sperm production. Nieschlag E et al. found that HCG monotherapy or HCG + HMG therapy was significantly more effective in restoring fertility in men with HH [17]. Although HCG + HMG therapy demonstrated superiority in promoting spermatogenesis and increasing sperm viability, it presented challenges in terms of administration, such as increased pain and poor compliance, and deviated from normal pituitary–gonadal physiology. GnRH micropumps deliver the pulsatile form of GnRH supplementation in vitro to elevate FSH, LH, and T levels, effectively promoting the development of external genitalia such as the penis and testes, increasing the frequency of morning erections, improving sperm survival, libido, and even greatly enhancing rates of spermatogenesis and sperm motility[18, 19]. Schopohl et al. revealed that patients with HH treated with a micropump pulse infusion of GnRH exhibited larger testicular volume and higher levels of estrogen and T compared to those treated with gonadotropins [20]. However, it should be noted that among the latter group, 5 patients developed breast development as a result of overstimulation-induced estrogen elevation. Huang B et al. discovered that pulse GnRH replacement or LH + FSH treatment could induce spermatogenesis, but pulse GnRH treatment resulted in earlier spermatogenesis, a greater number of spermatozoa, a higher spermatogenesis activity rate, and larger testicular volume [21]. Blumenfeld Z et al. switched 2 patients with HH who had unsuccessful gonadotropin treatment to pulsed GnRH treatment and ultimately achieved sperm production [22]. Nevertheless, it is important to acknowledge that the financial burden for patients significantly increases with pulsed GnRH micropump replacement, despite its closer adherence to the physiological regulation of the HPG axis. In this particular case, the patient who had secondary HH and developed breasts, expressed a desire to have offspring. After undergoing pulsed GnRH treatment, the patient's symptoms gradually improved, with hormone levels returning to normal and a significant increase in sperm motility and normal sperm count. Ultimately, his wife conceived naturally and gave birth to a son.

When to stop pulsed GnRH therapy is not a clinical standard. Theoretically, long-term pulsed GnRH replacement therapy is considered the most suitable approach for HH patients. HH patients who do not require immediate fertility can undergo GnRH micro-pumping treatment to obtain cryopreserved high-quality sperm. In cases where their partners have experienced prolonged infertility, advanced assisted reproductive techniques in reproductive medicine units may enable conception [23]. In cases where their partners have experienced prolonged infertility, advanced assisted reproductive techniques in reproductive medicine units may enable conception. Note that the risk of prostate cancer should be assessed by a urologist before androgen replacement therapy [24]. In this case, GnRH pulse replacement was discontinued one month after the patient's partner successfully became pregnant. To preserve male secondary sexual characteristics, the patient's low-dose T was adjusted due to factors such as the patient's age, risk of urological neoplasia, and absence of future reproductive intentions.

## Conclusion

The patient presented with secondary HH caused by PES, exhibiting hypogonadotropic signs and mammary development, along with decreased levels of FSH, LH, and T. Following treatment with GnRH pulse therapy, the patient's symptoms resolved, hormone levels returned to normal, and his wife achieved a healthy pregnancy. In patients with secondary hypogonadism, GnRH pulse therapy can be used to restore reproductive function.

## Data Availability

The datasets used and/or analyzed during the current study are available from the corresponding author upon reasonable request.
